# Characterization of glycogen-related glycoside hydrolase *glgX* and *glgB* from *Klebsiella pneumoniae* and their roles in biofilm formation and virulence

**DOI:** 10.3389/fcimb.2024.1507332

**Published:** 2024-12-18

**Authors:** Xinyue Liu, Jialin Li, Ruibing Wu, Liping Bai

**Affiliations:** ^1^ College of Basic Medicine, Inner Mongolia Medical University, Hohhot, China; ^2^ NHC Key Laboratory of Biotechnology of Antibiotics, Institute of Medicinal Biotechnology, Chinese Academy of Medical Sciences and Peking Union Medical College, Beijing, China

**Keywords:** glycogen, glycoside hydrolase, *Klebsiella pneumoniae*, enzyme activity, biofilm, virulence

## Abstract

Glycogen is a polymer used by bacteria to store excess glucose, playing a crucial role in bacterial growth, stress resistance, biofilm formation, and virulence. In bacteria, the glycoside hydrolase family 13 protein are involved in the synthesis and metabolism of glycogen, respectively. The absence of these enzymes leads to changes in bacterial glycogen content, thereby affecting the growth metabolism of the strain. To date, research on the roles of these glycogen-related glycoside hydrolase genes in the synthesis metabolism and bacterial phenotypes of *Klebsiella pneumoniae* has been limited. In this study, we characterized the glycogen-related glycoside hydrolase genes *glgB* and *glgX* of *K. pneumoniae*. We found that both enzymes exhibited significant degradation activity against glycogen substrates and were capable of degrading amylopectin, amylose, and pullulan. The optimal temperatures for GlgB and GlgX were both in the range of 35-40°C, with optimal pH values of 7.5 and 7.0, respectively, and they exhibited high stability at 37°C. Subsequently, we deleted the *glgB* and *glgX* genes in *K. pneumoniae*. The deletion of the *glgB* gene resulted in a decrease in the growth rate of the bacteria and defected glycogen synthesis. In contrast, the deletion of the *glgX* gene slightly accelerated the growth rate and led to continuous glycogen accumulation. In terms of biofilm formation and virulence, defects in glycogen synthesis impeded biofilm formation and virulence, while continuous glycogen accumulation did not affect biofilm formation but slightly increased virulence. In conclusion, the *glgB* and *glgX* genes are essential for the glycogen synthesis and metabolism in *K. pneumoniae* and further influence the biofilm formation capacity and virulence.

## 1 Introduction

Excessive glucose ingested by organisms is stored in the form of inactive polymers, with glycogen serving as such a polymer in bacteria (Wilson et al., 2010; Montero et al., 2011; Liu et al., 2021; Neoh et al., 2024). Glycogen is a polysaccharide with a highly branched structure, primarily composed of glucose residues linked by α-1,4-glycosidic bonds and α-1,6-glycosidic bonds (Li et al., 2023; Neoh et al., 2024). In both prokaryotes and eukaryotes, glycogen can be categorized into three classes based on diameter: protein-rich γ particles less than 3 nm, spherical β particles approximately 20 nm in size, and larger rose-like α particles (Li and Hu, 2020; Liu et al., 2021). There are differences in the utilization and degradation rates among glycogen particles of different levels (Besford et al., 2016). Currently, the process of glycogen synthesis in eukaryotes has been well studied, whereas some synthesis steps of glycogen in prokaryotes remain to be elucidated (Cifuente et al., 2019; Liu et al., 2021; Jenkins et al., 2023).

Glycogen primarily serves as a major energy reserve in prokaryotes, and current research indicates that it is closely associated with the organism’s tolerance to extreme environments, its ability to colonize hosts, and its virulence (Wang et al., 2015; Kwak et al., 2016). The synthesis and metabolism of glycogen in prokaryotes constitute a complex pathway involving a variety of genes responsible for synthesis, metabolism, and regulation (Wilson et al., 2010; Neoh et al., 2024). Among these, five key enzymes have been identified: glucose-1-phosphate adenylyltransferase (AGPase), glycogen phosphorylase (GP), glycogen synthase (GS), glycogen branching enzyme (GBE), and glycogen debranching enzyme (GDE), with both GBE and GDE belonging to the glycoside hydrolase family 13 (GH13) (Seibold et al., 2011; Machtey et al., 2012; Jenkins et al., 2023). In *Escherichia coli*, these enzymes are encoded by the genes *glgB* (GBE), *glgX* (GDE), *glgC* (AGPase), *glgA* (GS), and *glgP* (GP), respectively, and they are organized within an operon named *glgBXCAP*, collaboratively participating in the synthesis and metabolic processes of glycogen in the bacteria (Wang et al., 2020). The genes *glgP* and *glgX* are crucial for the formation of glycogen structure; the absence of *glgP* and *glgX* affects the morphology, structural stability, and chain length of glycogen (Yu et al., 1988; Li et al., 2023). Concurrently, the deletion of the two glycogen-degrading enzymes, *glgP* and *glgX*, leads to the continuous accumulation of glycogen within the bacterial cell (Wang et al., 2020). Conversely, the deletion of the three glycogen-synthesizing genes, *glgB*, *glgC*, and *glgA*, inhibits the synthesis of glycogen (Wang et al., 2015; Kwak et al., 2016).

*Klebsiella pneumoniae*, a member of the *Enterobacteriaceae* family, is a Gram-negative bacterium that colonizes the intestinal tract of the host and can cause life-threatening diseases under certain conditions (Tacconelli et al., 2018; Wyres and Holt, 2022). Consequently, *K. pneumoniae*, along with other strains such as *Acinetobacter baumannii*, is included in the World Health Organization’s priority list for the development of new antibiotics (Tacconelli et al., 2018). The virulence of *K. pneumoniae* is linked to its survival capabilities in extreme conditions, particularly its ability to form biofilms, which can assist the bacterium in colonizing the surfaces of cells and enhances the bacterium’s resistance to antibiotics and its virulence (Sauer et al., 2022; Li and Ni, 2023).

Studies have shown that the development of inhibitors targeting the GlgB protein can effectively suppress the virulence and pathogenicity of *Mycobacterium tuberculosis* (Dkhar et al., 2015). Additionally, the deletion of *glgB* and *glgX* in *E. coli* results in an increase in biofilm biomass and a reduction in the strain’s viability under extreme conditions, indicating their significant roles in bacterial virulence and survival in harsh environments (Wang et al., 2015, 2020). However, despite ranking as the third most prevalent pathogen responsible for antimicrobial resistance associated deaths, *K. pneumoniae* has not been extensively studied, particularly in terms of the function of glycogen in the formation of its biofilm and the development of its virulence (Murray et al., 2022). Here, we investigated the GH13 family protein GlgB and GlgX in *K. pneumoniae*, which are annotated in the UniProt database as glycogen branching enzyme and glycogen debranching enzyme, respectively. Our findings indicate that these enzymes are capable of degrading glycogen. Following this, we conducted a deletion of the *glgB* and *glgX* genes in *K. pneumoniae* to assess the impact on bacterial growth, glycogen accumulation, biofilm formation, and virulence attributes.

## 2 Methods

### 2.1 Strains and growth conditions

The strains, plasmids and oligonucleotide primers used in this study are shown in **Supplementary Table S1**. All strains except those containing the pCaskp plasmid were grown in liquid (shaking at 220 rpm) or solid LB medium at 37°C in incubator or shaker (Zhichu).

### 2.2 Cloning, expression, and purification of protein

The DNA sequences for *glgB* and *glgX* from *K. pneumoniae* MGH78578 (GCF_000016305.1) were retrieved from the GeneBank database respectively. Utilizing the genomic DNA of *K. pneumoniae* MGH78578 as a template, the *glgB* and *glgX* genes were amplified via PCR using the PrimerStar DNA Polymerase (Takara). Subsequently, the amplified *glgB* and *glgX* sequences were cloned into the pET-28a vector through the In-Fusion Snap Assembly Master Mix (Takara).

*E.coli* BL21 (DE3) cells (Transgen Biotech) containing pET28a-*glgB* or pET28a-*glgX* vector were inoculated into LB medium with kanamycin and incubated at 37°C until OD600 was 0.8, and then incubated at 16°C for 20 h with isopropyl β-D-1-thiogalactoside of 0.5 mM. Bacterial cells were resuspended with binding buffer (50 mM Tris, 300 mM NaCl, pH 7.5) and lysed by FastPrep-24™ 5G homogenizer (MP Biomedicals). Recombinant proteins were enriched by Ni-NTA resins and eluted with elution buffer (50 mM Tris, 500 mM Imidazole, 300 mM NaCl, pH 7.5). Fractions were concentrated by an Amicon Ultra-15 centrifugal filter (Millipore) with a molecular weight cutoff of 10 kDa.

### 2.3 Activity and properties of enzymes

The assessment of enzyme activity was modified based on previous study (Schumacher et al., 2022). Briefly, the enzyme activity is assessed by measuring the increase in reducing sugars in the reaction solution. After purification, the enzyme is mixed with phosphate buffer and a final concentration of 0.05% glycogen solution, and incubated at 37°C for one hour. Following incubation, an equal volume of bicinchoninic acid (BCA) reagent is added to the reaction mixture and vortexed for 60 seconds on a vortex mixer (IKA), followed by incubation at 80°C for 30 minutes. After the reaction solution has cooled to room temperature, 125 µL of the solution is transferred to a 96-well plate for the measurement of absorbance at 562 nm. D-glucose is used to create the standard curve, and the absorbance values at 562 nm are converted to reducing ends [µM glucose equivalents]. All measurements are performed in triplicate.

To determine the optimal temperature, the enzyme is placed in phosphate buffer at pH 7.5, and its activity is measured at different temperatures in increments of 5°C. For the determination of the optimal pH, the phosphate buffer is replaced with sodium dihydrogen phosphate (100 mM, pH 6.0-7.5) and tris(hydroxymethyl)aminomethane (50 mM, pH 8.5-9.0) buffer solutions, respectively, and the enzyme activity is measured at the optimal temperature. Regarding temperature stability, the enzyme is incubated at the optimal temperature and pH for durations ranging from 4 to 28 hours, after which the activity is assessed.

### 2.4 Deletion mutants

A dual-plasmid CRISPR/Cas9 system was employed to delete the *glgB* and *glgX* genes in *K. pneumoniae* MGH78578 (Wang et al., 2018). The kanamycin resistance gene in the pSGKP plasmid was replaced with a hygromycin B resistance gene to circumvent the kanamycin resistance of *K. pneumoniae* MGH78578. The pCasKP plasmid was first electroporated into *K. pneumoniae* MGH78578 and the correct transformants were selected on LB plates containing apramycin. Subsequently, the pSGKP plasmid carrying spacer sequences along with ssDNA or dsDNA homology arms was co-transformed into *K. pneumoniae* MGH78578 containing the pCasKP plasmid, which had been induced with L-arabinose. The transformants were then screened on plates containing both apramycin and hygromycin B to further validate the deletion of the *glgB* or *glgX* genes. Strains with successful deletion of *glgB* or *glgX* were cultured overnight at 37°C on plates containing 5% sucrose to cure the plasmids. Strains that lost the pSGKP plasmid alone were cultured on plates with 5% sucrose at 30°C for subsequent gene deletion, and the strain with the simultaneous deletion of *glgB* and *glgX* was named *ΔglgBX*.

### 2.5 Growth curves

To determine the growth rate of the strains, they were cultured overnight in LB medium and then inoculated at a 1% dilution into M63+ medium. The Microscreen HT growth curve analyzer (Jieling Instruments) was used for the measurement of growth curves. The culture medium was added to the 48-well deep-well plate in 1 mL per well and placed in the instrument for incubation at 37°C and 800 rpm. The growth was measured every 20 minutes for a continuous period of 24 hours. All reactions were performed in triplicate.

### 2.6 Glycogen/protein ratio assay

The M9 minimal medium was employed for the cultivation of various *K. pneumoniae*. After the bacterial cells were harvested by centrifugation, the glycogen/protein ratio was determined using the method described in previously study (Li et al., 2023). Bacteria cells were lysed by FastPrep-24™ 5G homogenizer (MP Biomedicals). After centrifugation, the supernatant was reacted with 60% KOH at 95°C for 2 hours. After treatment, the supernatant was divided into two equal parts, one of which was used to determine protein concentration using the BCA method, while the other was used for the detection of glycogen content. For the determination of glycogen content, pre-cooled ethanol was added to the samples, and the mixture was thoroughly vortexed to facilitate glycogen precipitation. The mixture was then placed at -20°C for 20 hours. The precipitates containing glycogen granules were collected by centrifugation at 4°C and then treated with ethyl alcohol to wash away impurities, with the washing procedure repeated three times. Thereafter, the supernatant was discarded, and the glycogen granules were placed at 60°C to dry any remaining moisture. All glycogen granules were then resuspended in a sodium acetate solution (50 mM, pH 4.8, containing 0.3 mg/mL amyloglucosidase) and incubated overnight at 37°C to completely convert the glycogen granules into glucose. The glucose content in the reaction system was measured using a glucose detection kit (Solarbio) to estimate the glycogen content and calculate the glycogen/protein ratio.

### 2.7 Biofilm formation ability assay

The measurement of biofilm biomass was performed using a modified method described in previous study (Haney et al., 2021). *K. pneumoniae* MGH78578 and three deletion mutants were incubated overnight and then diluted to 1% in M63+ medium. The diluted bacterial solution was incubated with 96-well plate at 37°C for 24h. After incubation, the 96-well plate was washed twice with deionized water to remove floating bacteria and medium. The biofilm of each well was stained with 200 μL of 0.1% (w/v) crystal violet for 15 min, and washed twice with deionized water. It was dissolved with 150 μL of 33% (v/v) glacial acetic acid for 15 min, and the absorbance at 600 nm was measured using BioTek 800 TS microplate detector (Agilent).

### 2.8 Confocal laser scanning microscopy

The bacterial cells were stained with SYTO-9 prior to observation with the confocal laser scanning microscope (CLSM), as described in previous studies with some modifications (Qiao et al., 2022). *K. pneumoniae* MGH78578 and deletion mutant strains were incubated overnight and then diluted to 1% in M63+ medium. The diluted bacterial solution was placed in Lab-Tek™ Chamber Slide (Thermo Scientific) at a volume of 400 µL per well and incubated statically at 37°C for 12 hours. After incubation, the medium was removed, and ddH_2_O was used to wash away the planktonic bacteria, with the washing procedure being repeated once. SYTO-9 dye was then added to wells and incubated in the dark for 20 minutes. After discarding the dye, the wells were washed to remove any residual dye. A Zeiss LSM 710 Confocal Laser Scanning Microscope (CLSM) was utilized to observe the stained biofilms. Subsequent to acquisition, the images were processed with ZEN 2009 software and subjected to analysis with COMSTAT 2.1 software for the quantification of biofilm biomass.

### 2.9 Determination of virulence of strains

The virulence of *K. pneumoniae* is assessed using *Galleria mellonella* larvae model (Schaefer et al., 2024). *G. mellonella* larvae were randomly divided into four groups, with ten in each group. Three groups were injected with 5 µL of wild type, *ΔglgB* and *ΔglgX* mutants, and the control group was injected with 5 µL of PBS solution. Larvae were incubated in an incubator at 37°C and 60% humidity. The survival of the larvae was observed from day 0 to day 4, with larvae that were unable to right themselves being considered deceased.

### 2.10 Statistical analysis

Statistical analyses were conducted utilizing GraphPad Prism software, wherein a one-way ANOVA was employed to assess the significance of differences. Each experimental was conducted with a minimum of three replicates to ensure reliability of the results.

## 3 Results

### 3.1 GlgB and GlgX catalyze the degradation of glycogen

**Figure 1:**
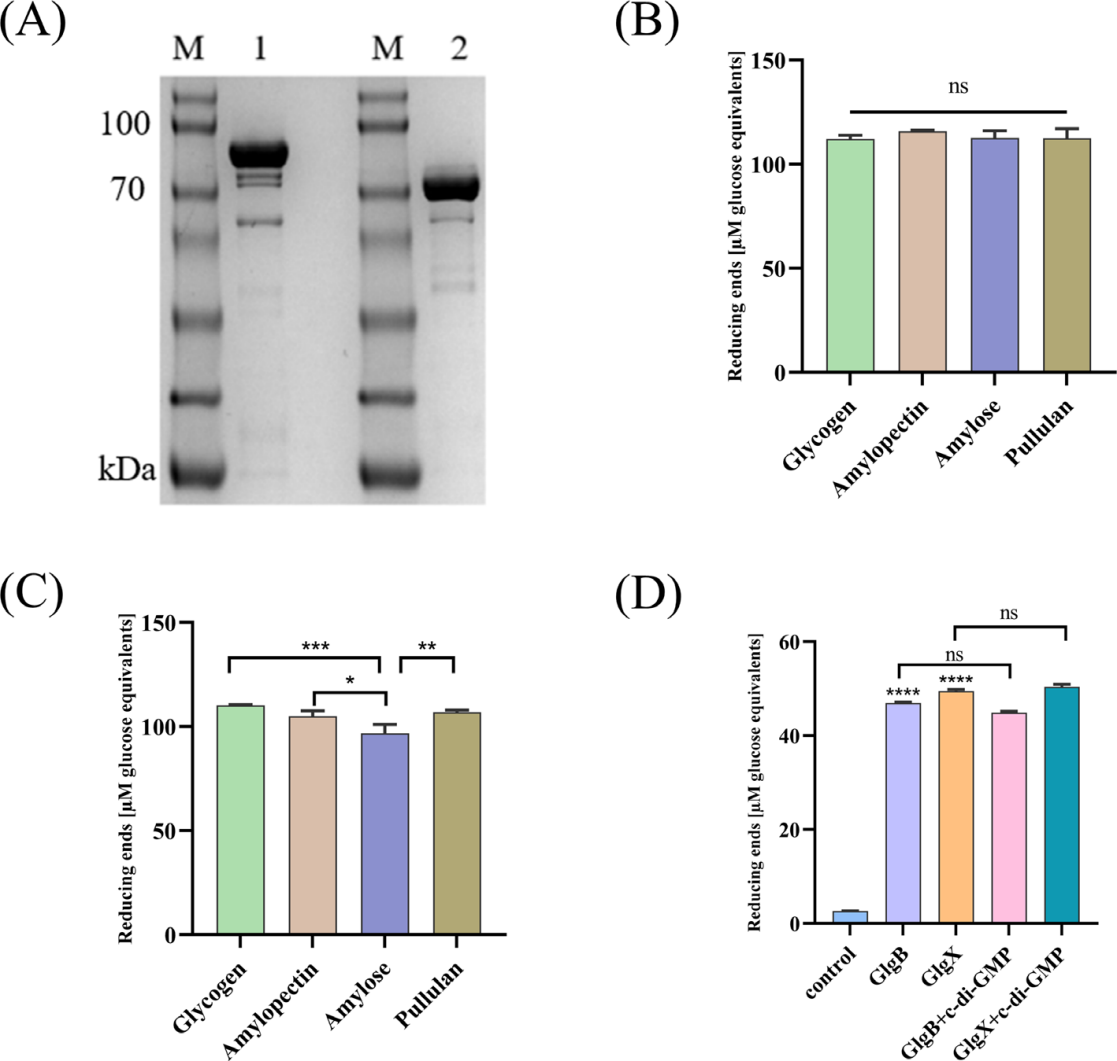
The activity of GlgB and GlgX. **(A)** Expression of GlgB and GlgX proteins in *E coli*. M-Protein marker, 1- GlgB protein, 2- GlgX protein. **(B)** The enzyme activity of GlgB towards different substrates. **(C)** The enzyme activity of GlgX towards different substrates. **(D)** Protein activity is not regulated by c-di-GMP. Error bars represent ± S.D of the mean. *p<0.05, **p<0.01, ***p<0.001, ****p<0.0001, ns indicates no statistical significance.

**Figure 2:**
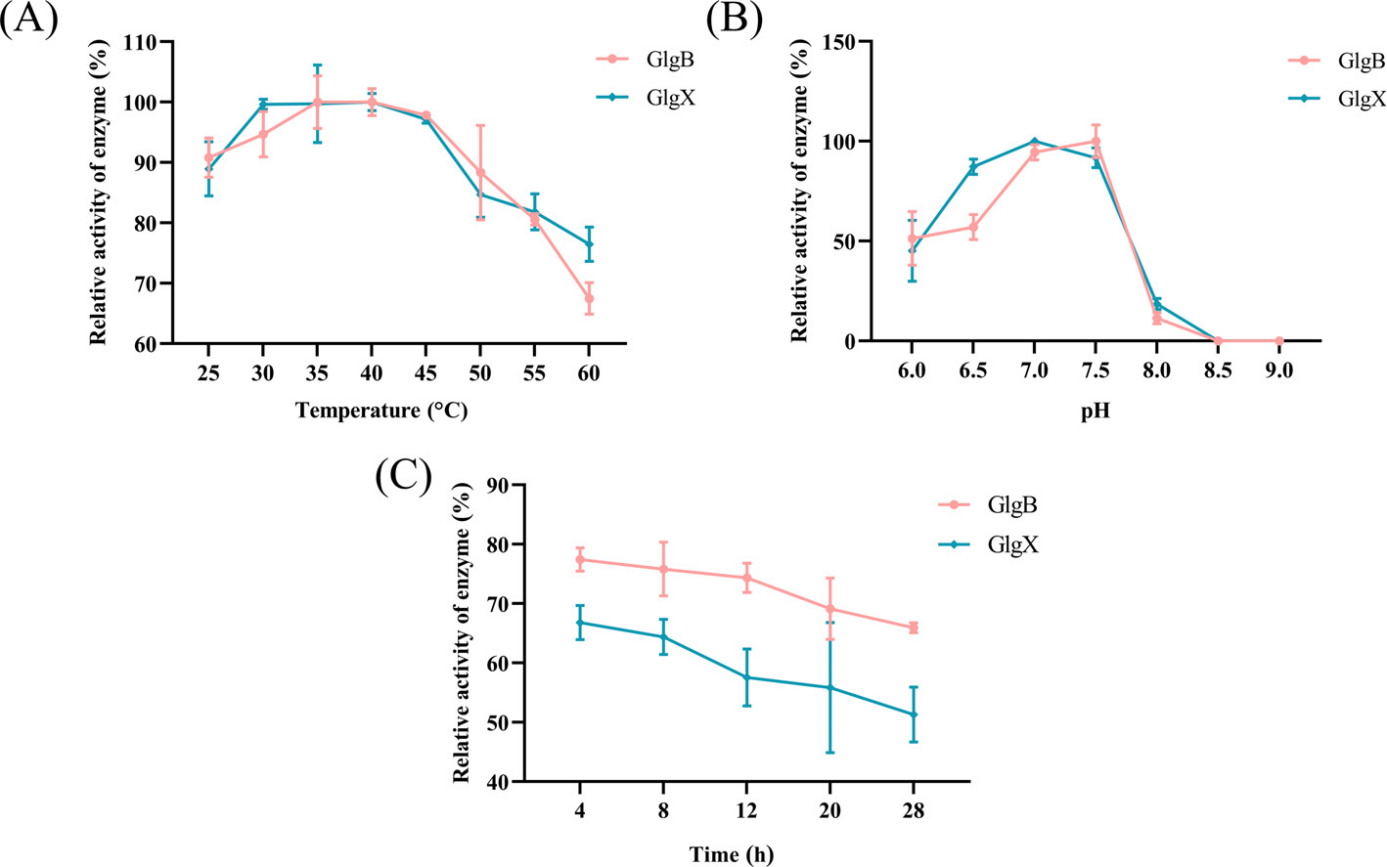
The enzyme properties of GlgX and GlgB. **(A)** Enzyme activity measured at different temperatures with the maximum activity group as 100%. **(B)** Enzyme activity measured at different pH values with the maximum activity group as 100%. **(C)** The residual activity of the enzyme after incubation from 4 to 28h using the enzyme activity at 0 h as 100%. Error bars represent ± S.D of the mean.

GlgX and GlgB both belong to the GH13 family, which contains an α-amylase domain, and they can catalyze the degradation of glycosidic bonds. The GlgX and GlgB proteins were expressed in *E. coli* and purified with Ni-NTA resin to obtain soluble proteins (**Figure 1A**). The degradation activities of GlgX and GlgB against glycogen, pullulan, amylose, and amylopectin were measured using the BCA method. We observed that GlgB catalyzed the hydrolysis of all substrates to a nearly equivalent extent (**Figure 1B**). In contrast, GlgX exhibits a pronounced substrate specificity, with the highest degradation activity towards glycogen and pullulan, while its activity towards amylose and amylopectin is significantly reduced (**Figure 1C**). Previous studies have indicated that Bis-(3′-5′)-cyclic dimeric GMP (c-di-GMP) enhances the hydrolytic activity of GlgX from *E. coli* towards glycogen (Schumacher et al., 2022). However, the addition of c-di-GMP at a final concentration of 50 μM did not increase the enzyme activity (**Figure 1D**). Subsequently, the enzymatic properties were characterized. Notably, both enzymes showed activity in a broad optimal temperature range, with peak activity observed between 35-40°C (**Figure 2A**). The optimal pH for GlgB was found to be 7.5, whereas for GlgX it was 7.0 (**Figure 2B**). When pH exceeding 7.5, the activity of both enzymes was markedly reduced (**Figure 2B**). To evaluate the stability of the enzymes, they were incubated at 37°C for 28 hours. A significant decrease in activity was noted after 4 hours of incubation, followed by a gradual decline in activity thereafter (**Figure 2C**). Two proteins have been shown to possess glycogenolytic activity *in vitro*, prompting us to further examine their activity *in vivo*.

### 3.2 The *glgB* and *glgX* genes alter glycogen content and affect the growth rate of the bacterium.

**Figure 3:**
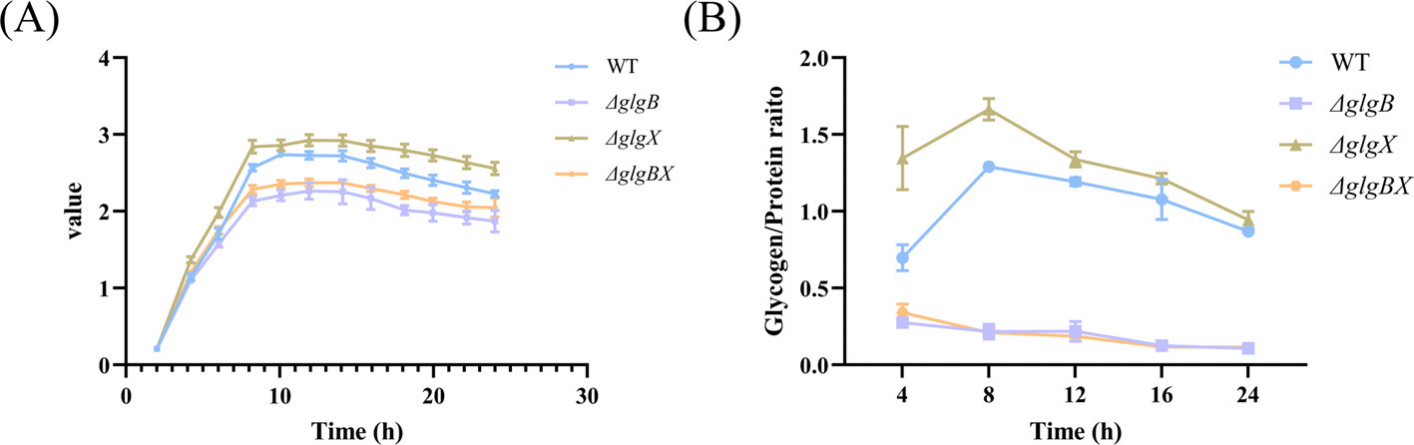
Changes in growth rate and glycogen content of wild-type *K. pneumoniae* and mutant strains. **(A)** Growth curves of WT and deletion mutations. **(B)** The glycogen content of WT and deletion mutations. Error bars represent ± S.D of the mean.

GlgB and GlgX are annotated as glycogen branching enzyme and debranching enzyme, respectively, in the UNIPROT database. It has been evidenced that these enzymes play a crucial role in bacteria, such as bacterial growth and the synthesis of glycogen (Wang et al., 2020). To investigate the role of protein in *K.pneumoniae*, we deleted the active center of GlgX and GlgB, resulting in the *ΔglgX*, *ΔglgB*, and *ΔglgBX* mutants (**Supplementary Figure S1**). Analysis of growth curves revealed that the *ΔglgX* mutant exhibited an accelerated growth rate, while the *ΔglgB* mutants and the *ΔglgBX* mutant displayed a slower growth pattern compared to the wild-type strain (**Figure 3A**). As glycogen serves as a crucial energy reserve for bacterial growth, the growth rate of a strain may be influenced by its glycogen content. Therefore, we quantified glycogen levels at different times throughout the bacterial growth cycle. The glycogen content in both the wild-type and *ΔglgX* mutants reached a peak at 8 hours post-inoculation and subsequently decreased, with the *ΔglgX* mutant maintaining a slightly higher glycogen level than the wild type (**Figure 3B**). The glycogen content in the *ΔglgB* and *ΔglgBX* mutants was comparable and remained relatively stable during the growth phase, consistently lower than that of the wild type (**Figure 3B**). The correlation between glycogen content and growth rate among the strains suggests that the amount of glycogen present impacts the growth rate of the bacterial strains. Additionally, since the *glgB* gene is located upstream of the *glgX* gene, which leads to the phenotypic similarity between *ΔglgB* and *ΔglgBX* mutants, future research will be directed exclusively towards investigating the *ΔglgB* and *ΔglgX* mutant.

### 3.3 Only the *glgB* gene influences biofilm formation of *K. pneumoniae*

**Figure 4:**
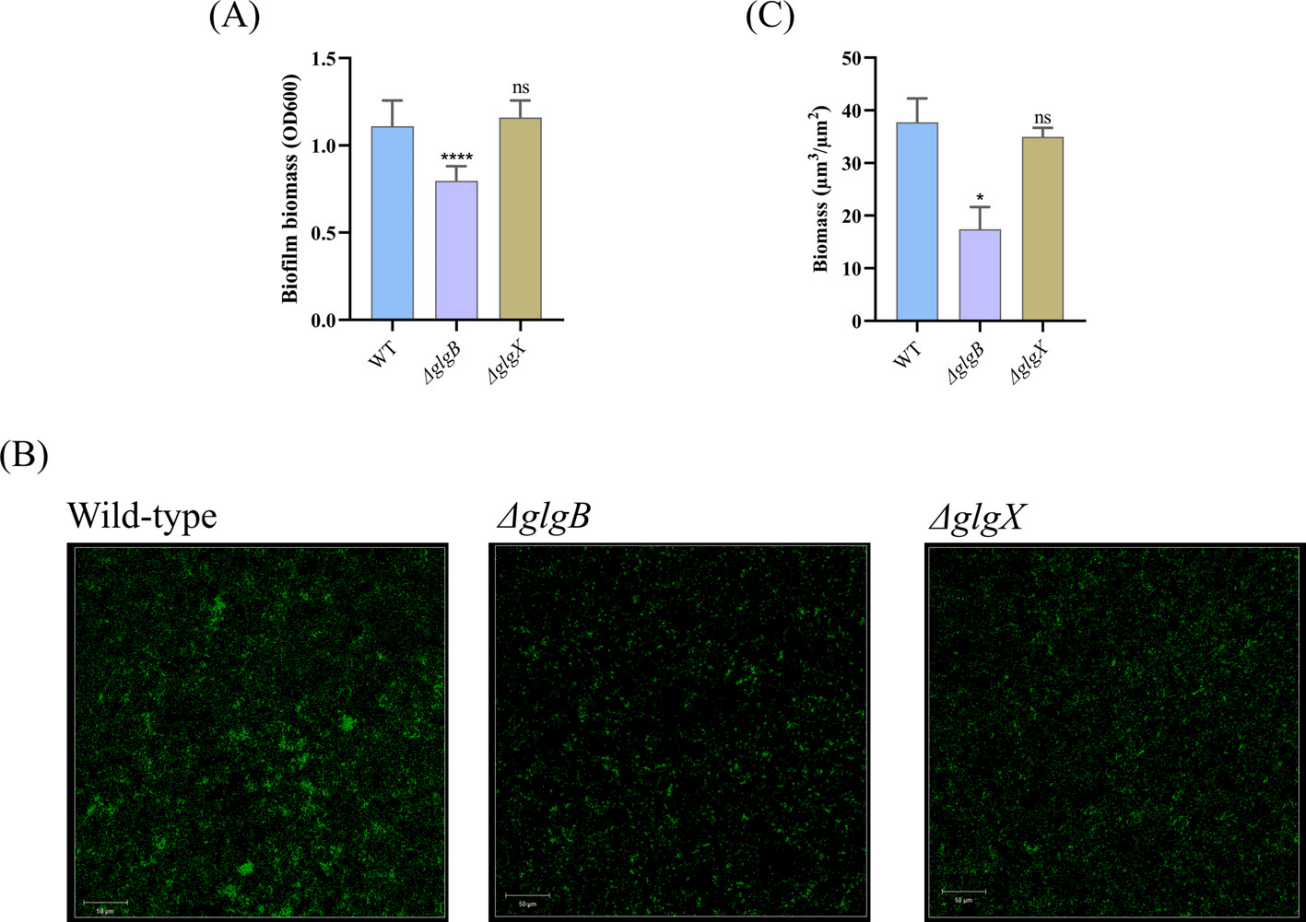
Biofilm biomass of wild-type *K. pneumoniae* and mutant strains. **(A)** Determination of biofilm biomass in WT and deletion mutations. **(B)** Representative confocal images of *K. pneumoniae* and deletion mutants. Scale bar, 50 μm. **(C)** The biofilm biomass of confocal images quantified by COMSTAT 2.1 software. Error bars represent ± S.D of the mean. *p<0.05, ****p<0.0001, ns indicates no statistical significance.

Previous study has indicated that bacterial glycogen can influence biofilm formulation (Wang et al., 2020). To quantify the impact of glycogen on biofilm formation, we utilized crystal violet staining to assess biofilm biomass. The data revealed a reduction in biofilm biomass following the deletion of the *glgB* gene, while the deletion of the *glgX* gene did not alter biofilm biomass (**Figure 4A**). To further investigate the effects of glycogen metabolism on biofilm formation, we employed CLSM and utilized SYTO9 stain the bacterial cells, respectively. In the corresponding images, both *ΔglgX* and *ΔglgB* mutants showed a decrease in bacterial biomass compared to the wild type, with the *ΔglgB* mutant showing a greater decrease in biomass than the *ΔglgX* mutant (**Figure 4B**). Subsequently, the COMSTAT software was employed to quantify the bacterial biomass within the images and to assess the significance of the biomass reduction. We found that the biomass of the *ΔglgB* mutant was significantly lower than that of the wild type, whereas the decrease in biomass of the *ΔglgX* mutant was not significant, indicating that the deletion of *glgB* more profoundly affects the biofilm formation capacity of *K. pneumoniae* (**Figure 4C**). This indicates that the deletion of *glgB* reduces the biofilm-forming ability of *K. pneumoniae*. 

### 3.4 The *glgB* and *glgX* genes can influence the virulence of *K. pneumoniae*

**Figure 5:**
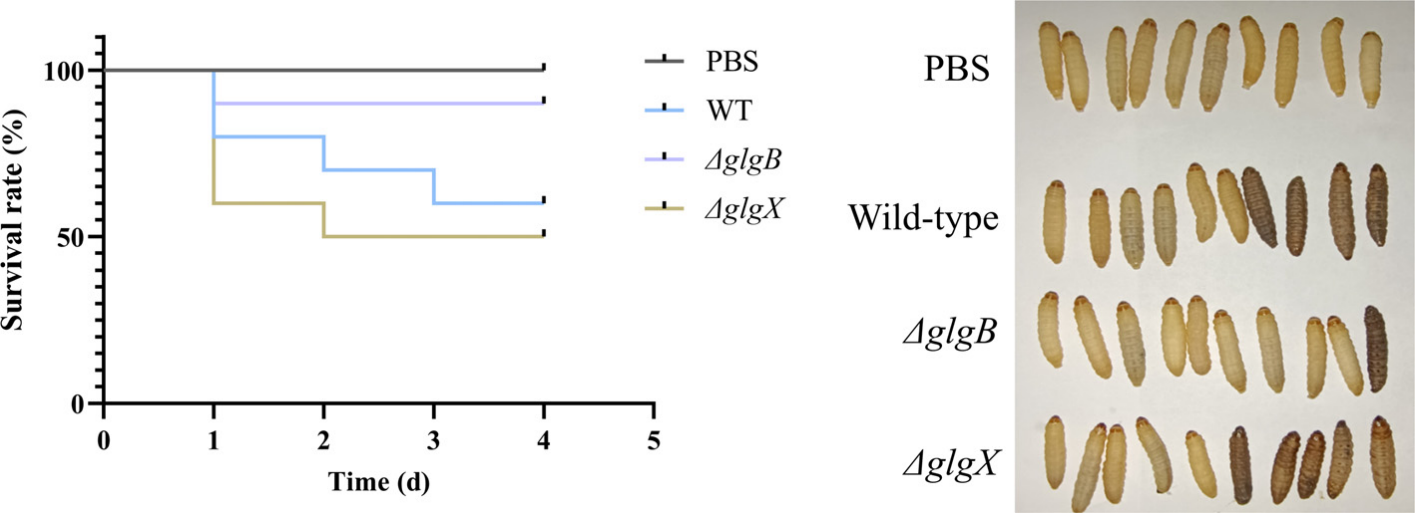
Survival rate of *G. mellonella* larvae infected with wild-type *K. pneumoniae* or mutant strains, PBS group as control.

The deletion of *glgB* and *glgX* genes in bacteria resulted in alterations to the growth rate and biofilm biomass compared to the wild type, leading us to hypothesize that these changes might also affect the virulence of the bacteria. Given that the *G. mellonella* larval model has been widely used in numerous studies to assess bacterial virulence and the efficacy of antimicrobial agents, we adopted this model to assess the virulence profiles of distinct *K. pneumoniae* isolates (Jin et al., 2024; Firacative et al., 2020). Following the inoculation of larvae with 1×10^6^ CFU of the respective *K. pneumoniae* strains, a marked difference in survival rates was observed. Larvae injected with the *ΔglgB* mutant strain exhibited a consistent survival rate of 90% from the initial to the fourth day post-inoculation (**Figure 5**). In contrast, the survival rate of larvae exposed to the wild-type strain progressively declined from 80% to 60% over the same period, signifying a reduced virulence in the *ΔglgB* mutant strain compared to the wild-type (**Figure 5**). Notably, the survival rate of larvae inoculated with the *ΔglgX* mutant strain decreased from 60% to 50% between the initial day and the fourth day following inoculation, implying a virulence slightly higher than the wild-type strain (**Figure 5**). Considering that the biofilm formation capacity of the *ΔglgB* strain is significantly reduced compared to the wild-type strain, this suggests that the absence of glycogen in bacteria leads to a decrease in key factors such as biofilm formation, which in turn diminishes the virulence of the organism.

## 4 Discussion

According to the InterPro database, GlgX and GlgB can catalyze the degradation of α-1,6 glycosidic bonds and α-1,4 glycosidic bonds, respectively. To date, GlgX has been reported to degrade various substrates, including glycogen and pullulan (Schumacher et al., 2022). However, the degradation activity of GlgB towards different substrates still requires investigation. After characterizing the GlgB and GlgX proteins from *K. pneumoniae*, we found that the GlgX protein exhibited the expected significant activity as expected. Concurrently, the GlgB protein also demonstrated considerable activity against various substrates, indicating that GlgB is capable of degrading α-1,4 glycosidic bonds in different substrates and producing glucose residues.

Previous studies have demonstrated that GlgX derived from *E. coli* and *Streptomyces* exhibits a significant enhancement in degradation activity towards various substrates in the presence of c-di-GMP (Schumacher et al., 2022; Gallagher et al., 2024). However, we did not observe the promotional effect of c-di-GMP on the activity of GlgX derived from *K. pneumoniae*. Considering that GlgX proteins with different structures have been shown to exhibit significant differences in activity, this suggests that the promotional effect of c-di-GMP on enzyme activity is limited to specific strains (Song et al., 2010). There are differences in the degradation capabilities of GlgX from various sources towards different polysaccharide substrates. The substrate specificity of GlgX derived from *K. pneumoniae* is similar to that of GlgX from *E. coli*, whereas differ with GlgX derived from *Vibrio cholerae* (Schumacher et al., 2022; Han et al., 2022).

In the synthesis and metabolism of glycogen in prokaryotes, the glycogen branching enzyme GlgB forms branches in the glycogen polymer by linking glucose residues through α-1,6-glycosidic bonds, forming a glucan branch, while the GlgX protein catalyzes the hydrolysis of glucose residues in the α-1,6-glycosidic bonds (Rashid et al., 2016; Strydom et al., 2017; Neoh et al., 2024). The deletion of *glgB* in *E. coli* impedes glycogen synthesis, while the deletion of *glgX* leads to excessive glycogen accumulation, which indirectly affects the growth rate of *E. coli* and its tolerance to extreme environments (Wang et al., 2020). Studies have demonstrated that excessive glycogen accumulation can enhance the growth rate of bacterial cells (Wang et al., 2020; Okabe et al., 2021). Conversely, the deficiency of glycogen results in a reduced growth rate of the bacterial cells (Lee et al., 2021). The deletion of *glgB* and *glgX* genes in *K. pneumoniae* corresponds to the defects observed in *E. coli*, leading to deficiencies in glycogen synthesis and degradation in *K. pneumoniae*, which in turn affects the growth rate of the strain. This evidence confirms that glycogen accumulation is essential for the growth and metabolic processes of *K. pneumoniae*.

The formation of biofilms is a strategy used by specific pathogens to withstand environmental stresses and resist antibiotics (Rumbaugh and Sauer, 2020; Akbarian et al., 2022; Lin et al., 2022). Mature biofilms provide pathogens with moisture and nutrients, impede the penetration of antibiotics, and facilitate immune evasion by bacteria (Rumbaugh and Sauer, 2020; Vandana and Das, 2022). The formation of biofilms is regulated by numerous factors, including the intake of nutrients, environmental conditions, and specific signaling molecules (Flemming et al., 2023). Glucose has been identified as an influence on the biofilm formation of *Staphylococcus epidermidis*, while the impact of glycogen on bacterial biofilm formation remains inconclusive (Lerner et al., 2009; Ahmad et al., 2022). It has been reported that the deletion of both *glgX* and *glgB* genes enhances the biofilm formation in *E. coli*, whereas studies have also indicated that inhibiting glycogen synthesis can decrease biofilm formation in *Salmonella enteritidis* (Bonafonte et al., 2000; Wang et al., 2020). The glycogen synthesis defect caused by the deletion of *glgB* does indeed inhibit the formation of biofilms in *K. pneumoniae*. However, the increase in biofilm biomass observed in the *ΔglgX* mutant of *E. coli* was not occur in *K. pneumoniae*, which may be due to differences in the complexity of biofilms between different bacteria (Wang et al., 2020).

The glycogen content in bacterial cells has also been demonstrated to be closely associated with their virulence (Tan et al., 2022; Miao et al., 2023). Multiple studies have shown that inhibiting the function of *glgB* can effectively reduce the virulence of *M. tuberculosis*, whereas the deletion of *glgX* has been associated with an increase in the virulence of *V. cholerae* (Dkhar et al., 2015; Han et al., 2022). In our evaluation of the virulence of *K. pneumoniae* using the *G. mellonell*a larval model, we demonstrated that the deletion of *glgB* reduces the virulence of the bacterium, indicating a positive correlation between glycogen content and the virulence of pathogenic bacteria.

Glycogen has been demonstrated to be closely associated with the virulence of pathogens, and inhibiting glycogen synthesis can effectively reduce the virulence of certain pathogenic bacteria. Our research indicates that the GlgX protein derived from *K. pneumoniae* is capable of efficiently degrading glycogen. Notably, the GlgB protein also exhibits activity against glycogen substrates, highlighting its potential application prospects. Additionally, this study constructed *ΔglgX* and *ΔglgB* mutants of *K. pneumoniae*, indicating that the deletion of *glgB* and *glgX* genes alters the bacterial growth rate by participating in glycogen synthesis and metabolism, thereby affecting the biofilm biomass and the virulence of the *K. pneumoniae*. Our research reveals the significant role of glycogen synthesis and metabolism in the formation of *K. pneumoniae* biofilms and its virulence, providing a strategy for addressing severe infections caused by this pathogen.

## Data Availability

The raw data supporting the conclusions of this article will be made available by the authors, without undue reservation.
