# Supplementary material for: Characterization of glycogen-related glycoside hydrolase glgX and glgB from Klebsiella pneumoniae and their roles in biofilm formation and virulence
Source: Front Cell Infect Microbiol. 2024 Dec 18;14:1507332. doi: 10.3389/fcimb.2024.1507332 (PMC11688210; doi:10.3389/fcimb.2024.1507332)
Supplement: Supplementary file 1 [file Table1.docx]

***Supplementary Material***

**Supplementary Figures and Tables**


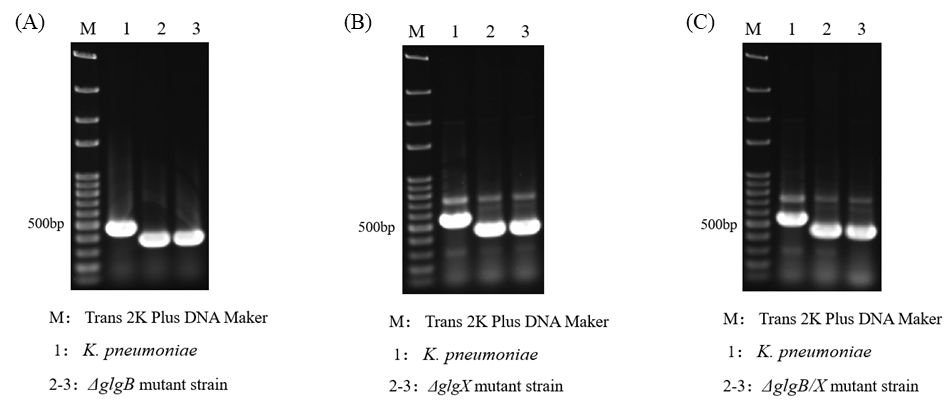


Supplementary Figure 1: Gene knockout validation.

A. The *glgB* gene validation.

B. The *glgX* gene validation.

C. The *glgB* and *glgX* genes validation.

**Table S1.** Strains, plasmids and oligonucleotide primers used in this study.

| Bacterial strains | | |
| --- | --- | --- |
| Name | Source |  |
| *Klebsiella Pneumoniae* MGH78578 | ATCC |  |
| *ΔglgB* mutant | This work |  |
| *ΔglgX* mutant | This work |  |
| *ΔglgBX* mutant | This work |  |
| *Escherichia coli* DH5α | Transgen Biotech |  |
| *Escherichia coli* BL21 | Transgen Biotech |  |
| Plasmids | | |
| Name | Source |  |
| pET28a-glgB | This work |  |
| pET28a-glgX | This work |  |
| pCasKP | Addgene |  |
| pSGKP | Addgene |  |
| Oligonucleotides | | |
| Name | Application | Squence (5´-3´) |
| GlgB-F | Amplification of the glgB gene | CGCGCGGCAGCCATATGTCTAATCATATTGATAGAGACG |
| GlgB-R | Amplification of the glgB gene | GGTGGTGGTGCTCGAGTTATTGCGCCTCCCGGACC |
| GlgX-F | Amplification of the glgX gene | CGCGCGGCAGCCATATGACCTCACTCGCGGCAGGC |
| GlgX-R | Amplification of the glgX gene | GGTGGTGGTGCTCGAGTTACGACCTTTGAAATACGCACAC |
| Gxsg | sgRNA spacer for glgX | TAGTTCGACCTCGCCTCAGTGATG |
| Gxsg | sgRNA spacer for glgX | AAACCATCACTGAGGCGAGGTCGA |
| ssDNA | Repair template for glgX deletion | GCCCGGACCGATATCCCAGGGTTCCGCAATCAGTTTCACCTGCGAAAAGCCGTCGACATGGCACTCATCCACCCAAAAGCGCAAACACTG |
| Gxtest F | Detection of glgX deletions | GTGCTCAACCATAGTGCGGAAATC |
| Gxtest R | Detection of glgX deletions | AAATCCCCCAGGGAGACATTTTGC |
| GbSg F | sgRNA spacer for glgB | TAGTTCGCGATTACAGCCGCAAGG |
| GbSg R | sgRNA spacer for glgB | AAACCCTTGCGGCTGTAATCGCGA |
| Gbhomology-1F | Amplification of the glgB homologous arms | CGAACCAGTTTGATGCCCCG |
| Gbhomology-1R | Amplification of the glgB homologous arms | AATGGCTTCCAGCAGCGCATCGATGCCGAA |
| Gbhomology-2F | Amplification of the glgB homologous arms | ATCGATGCGCTGCTGGAAGCCATTGAGTTTCTGCG |
| Gbhomology-2R | Amplification of the glgB homologous arms | ACGCCGTGGTGCCAGTTGTC |
| Gbtest F | Detection of glgB deletions | AACGTCATTCTTGACTGGGTGCCC |
| Gbtest R | Detection of glgB deletions | GGTCATTTTGTCATGATGGTAGCGGC |
